# The Association of Dialysis Facility Payer Mix With Access to Kidney Transplantation

**DOI:** 10.1001/jamanetworkopen.2023.22803

**Published:** 2023-07-11

**Authors:** David C. Cron, Thomas C. Tsai, Rachel E. Patzer, Syed A. Husain, Lingwei Xiang, Joel T. Adler

**Affiliations:** 1 Department of Surgery, Massachusetts General Hospital, Boston; 2 Center for Surgery and Public Health, Brigham and Women’s Hospital, Boston, Massachusetts; 3 Department of Health Policy and Management, Harvard T.H. Chan School of Public Health, Boston, Massachusetts; 4 Department of Surgery, Emory University School of Medicine, Atlanta, Georgia; 5 Department of Medicine, Emory Medical School, Atlanta, Georgia; 6 Department of Medicine, Division of Nephrology, Columbia University Medical Center, New York, New York; 7 The Columbia University Renal Epidemiology Group, New York, New York; 8 Division of Transplantation, Department of Surgery and Perioperative Care, Dell Medical School at the University of Texas at Austin, Austin

## Abstract

**Importance:**

Insurance coverage for patients with end-stage kidney disease has shifted toward more commercially insured patients at dialysis facilities. The associations among insurance status, facility-level payer mix, and access to kidney transplantation are unclear.

**Objective:**

To determine the association of dialysis facility commercial payer mix and 1-year incidence of wait-listing for kidney transplantation, and to delineate the association of commercial insurance at the patient vs facility level.

**Design, Setting, and Participants:**

This retrospective population-based cohort study used data from the United States Renal Data System from 2013 to 2018. Participants included patients aged 18 to 75 years initiating chronic dialysis between 2013 and 2017, excluding patients with a prior kidney transplant or with major contraindications to kidney transplant. Data were analyzed from August 2021 and May 2023.

**Exposure:**

Dialysis facility commercial payer mix, calculated as the proportion of patients with commercial insurance per facility.

**Main Outcomes and Measures:**

The primary outcome was patients added to a waiting list for kidney transplant within 1 year of dialysis initiation. Multivariable Cox regression, censoring for death, was used to adjust for patient-level (demographic, socioeconomic, and medical) and facility-level factors.

**Results:**

A total of 233 003 patients (97 617 [41.9%] female patients; mean [SD] age, 58.0 [12.1] years) across 6565 facilities met inclusion criteria. Participants included 70 062 Black patients (30.1%), 42 820 Hispanic patients (18.4%), 105 368 White patients (45.2%), and 14 753 patients (6.3%) who identified as another race or ethnicity (eg, American Indian or Alaskan Native, Asian, Native Hawaiian or Pacific Islander, and multiracial). Of 6565 dialysis facilities, the mean (SD) commercial payer mix was 21.2% (15.6 percentage points). Patient-level commercial insurance was associated with increased incidence of wait-listing (adjusted hazard ratio [aHR], 1.86; 95% CI, 1.80-1.93; *P* < .001). At the facility-level and before covariate adjustment, higher commercial payer mix was associated with increased wait-listing (fourth vs first payer mix quartile [Q]: HR, 1.79; 95% CI, 1.67-1.91; *P* < .001). However, after covariate-adjustment, including adjusting for patient-level insurance status, commercial payer mix was not significantly associated with outcome (Q4 vs Q1: aHR, 1.02; 95% CI, 0.95-1.09; *P* = .60).

**Conclusions and Relevance:**

In this national cohort study of patients newly initiated on chronic dialysis, although patient-level commercial insurance was associated with higher access to the kidney transplant waiting lists, there was no independent association of facility-level commercial payer mix with patients being added to waiting lists for transplant. As the landscape of insurance coverage for dialysis evolves, the potential downstream impact on access to kidney transplant should be monitored.

## Key Points

**Question:**

Is dialysis facility–level commercial payer mix associated with patient access to kidney transplant?

**Findings:**

In this cohort study including 233 003 patients receiving incident dialysis, commercial insurance at the patient-level was associated with increased wait-listing for kidney transplant within 1 year, but there was no significant independent association of facility-level commercial payer mix with wait-listing.

**Meaning:**

These findings suggest that while patient insurance status was associated with access to kidney transplant, dialysis facility payer mix did not itself have an association with wait-listing; nevertheless, as the landscape of insurance coverage for dialysis evolves, the potential downstream impact on access to kidney transplant should be monitored.

## Teaser

This cohort study assesses the association of dialysis facility–level commercial payer mix with 1-year incidence of patient wait-listing for kidney transplant.

## Introduction

Nearly 800 000 patients are living with end-stage kidney disease (ESKD) in the United States.^1^ Kidney transplantation offers a survival benefit over dialysis, yet only 21% of patients with incident ESKD are wait-listed for transplant or receive transplant within 5 years of starting dialysis, and rates have been stagnant over the past 20 years.^1^ For most patients, access to kidney transplant starts with the dialysis facility via counseling and a referral for transplant evaluation, yet referral and wait-listing rates for kidney transplant vary widely across dialysis centers.^2^ As such, dialysis facility practices are increasingly the focus of health policy aimed at improving access to kidney transplantation.^3^

Dialysis reimbursement and dialysis facility practices related to patient insurance coverage have been scrutinized in recent years. Commercial insurance reimburses for dialysis at 4 times the rate of Medicare,^4^ and with increased availability of individual market coverage via the Affordable Care Act marketplaces,^5^ the payer mix at dialysis facilities has shifted toward more commercially insured patients.^6,7^ These payer mix trends have raised concerns among clinicians and policy makers about the potential for decreased access to kidney transplantation, but this has not been studied, to our knowledge.^5^ Facilities with a higher commercial payer mix, and thus higher reimbursement and revenue, may have greater ability to invest in transplant education and access, but the link between payer mix and kidney transplant access is unclear. At the individual patient level, commercial insurance can facilitate access to specialty care, and patients with commercial insurance are more likely to be evaluated, added to waiting lists, and undergo transplant than patients with noncommercial insurance.^8,9,10^ Understanding whether access to the transplant waiting list is associated with an individual patient-level contribution of insurance, such as the physician network or patient’s own resources, or to a facility-level factor, such as policies, practices, or culture to educate and refer patients to transplantation or aid them in navigating this process, is necessary to understand the ramifications of recent payer mix trends.^6,7^

To this end, we studied the association of dialysis facility commercial payer mix with patient access to kidney transplantation. We hypothesized that a higher dialysis facility–level commercial payer mix is associated with higher incidence of kidney transplant wait-listing within 1 year of dialysis initiation, even after accounting for individual patients’ insurance status.

## Methods

### Data Sources and Study Cohort

This cohort study was approved by the Mass General Brigham institutional review board with a waiver of informed consent because this was a retrospective analysis of deidentified data contained in a national registry. This study follows the Strengthening the Reporting of Observational Studies in Epidemiology (STROBE) reporting guideline. We used the United States Renal Data System (USRDS),^1^ a national data set of all patients with ESKD regardless of insurance provider. We obtained dialysis facility characteristics from the Medicare Dialysis Facility Compare.^11^ We included adult patients aged 18 to 75 years who initiated chronic dialysis between 2013 and 2017 and had 1 year of follow-up. Both hemodialysis (in-center and home) and peritoneal dialysis were included, as all patients are affiliated with a dialysis facility regardless of modality. We excluded patients with a prior kidney transplant to obtain a more homogenous cohort with respect to transplant candidacy. To further limit the cohort to reasonable transplant candidates, we excluded patients with potential major contraindications to transplant, including institutionalization (nursing home, assisted living, other), inability to transfer, drug or alcohol dependence, cancer, and those who were deemed unfit for or declined transplant referral (eFigure 1 in Supplement 1).

### Variables

Patient insurance at the time of dialysis initiation was ascertained using the Centers for Medicare & Medicaid Services (CMS) Medical Evidence Form 2728. This form is submitted for all patients with incident ESKD within 45 days of dialysis initiation and is commonly used for reporting demographic information for administrative, billing, and research purposes.^12^ For our primary exposure variable, patient insurance at dialysis initiation (ie, for patients with incident ESKD) was aggregated within each dialysis facility and we calculated the mean over the study period to compute facility commercial payer mix to find the proportion of patients at each facility with commercial insurance. This measure is thus reflective of the payer mix among new patients initiating dialysis care at each facility. Approximately 70% of patients with commercial insurance keep their insurance for the first 30 months through the coordination period,^12^ but some switch earlier to Medicare. We did not measure or account for the switching of insurance beyond initial enrollment. On Form 2728, multiple insurance options can be reported. If patients had commercial insurance (whether employer sponsored or obtained through the individual market), regardless of whether another insurance type was also reported, this was counted as commercial insurance for our purposes, since the commercial insurer is still likely to be the primary payer for dialysis in this setting. All other forms of insurance were considered noncommercial, including Medicare, Medicare Advantage (MA), Medicaid, Department of Veteran Affairs, or other. For analysis, payer mix was categorized into quartiles, with the first quartile (Q1) being lowest proportion of commercial payers and the fourth quartile (Q4), the highest.

Our primary outcome was time until addition to the kidney transplant waiting list up to 1 year after dialysis initiation (patient-level). This outcome was chosen in line with existing federal policy priorities and quality measures.^13^ We censored for death within 1 year of listing. Covariates were chosen by clinical and policy significance. Patient-level covariates included age, sex, race and ethnicity, employment status, insurance (commercial, Medicare, Medicaid, other), county-level Social Vulnerability Index (SVI; per 2016 data),^14,15^ initial dialysis modality (in-center hemodialysis, home hemodialysis, peritoneal), primary kidney disease cause, comorbidities, and whether the patient was informed about transplantation at dialysis initiation. Race and ethnicity were available from Form 2728 (collected by patient report or provided by patient’s facility) and was included in this study due to the known associations of race and ethnicity with disparities in access to kidney transplant. The SVI ranges from 0, indicating least vulnerable, to 1, most vulnerable. Facility-level covariates included size, location (urban, rural, micropolitan), for-profit status, and dialysis chain affiliation (Fresenius or DaVita). Additional facility characteristics, including patient-to-nurse ratio, patient-to-social worker ratio, and Dialysis Facility Compare (DFC) rating,^13^ were reported for descriptive purposes but were not included for covariate adjustment since they potentially exist on the causal pathway between exposure and outcome. The DFC rating ranges from 1 to 5 stars, with 1 indicating worst and 5, best.

### Statistical Analysis

Descriptive characteristics of the facilities and study cohort were computed across quartiles of payer mix. Continuous variables were summarized with mean and SD and categorical variables with frequency and percentage. The highest vs lowest payer mix quartiles were compared using *t* test, Fisher exact test, or χ^2^ test.

To estimate the independent association of dialysis facility commercial payer mix with kidney transplant wait-listing, we used multivariable Cox regression. The model was run at the patient-level with sandwich estimators for SEs to account for patients clustering within facilities. Because payer mix, a facility-level characteristic, is inherently related to the insurance status of individual patients in each facility, we performed and reported the model results in a stepwise fashion as follows, in attempt to delineate the patient-level association of commercial insurance for individuals vs the facility-level association of high commercial payer mix (and the higher reimbursement that follows) for dialysis facilities. We first computed the unadjusted model, then adjusted for all covariates except individual insurance status, and finally adjusted for all covariates including individual insurance status. This allows us to qualitatively compare the association of payer mix with outcome before and after accounting for the contribution of insurance at the patient-level. Finally, we included an interaction term (payer mix by patient-level insurance) to test if the association of commercial payer mix with the hazards of wait-listing differed for patients with commercial vs noncommercial insurance. If commercial payer mix is associated with outcome at the facility-level (ie, better performance by facilities with high payer mix and thus greater revenue), we would expect an association of payer mix with outcome even in the subset of publicly insured patients at facilities with higher payer mix.

We performed sensitivity analyses to assess consistency of our results among patients with higher likelihood of being added to transplant waiting lists. We repeated analyses among a subset of patients aged 65 years or younger. Then, we repeated analyses in the age 65 years or younger cohort while also excluding patients with more relative contraindications to transplantation (in addition to the major contraindications excluded from the main analysis): congestive heart failure, chronic obstructive pulmonary disease, peripheral vascular disease, amputation, nonambulatory status, or requiring assistance with activities of daily living.

Two-tailed tests with an α = .05 were used for statistical significance testing. All analyses were performed using Stata statistical software version 14.1 (StataCorp) between August 2021 and May 2023.

## Results

### Commercial Insurance and Commercial Payer Mix

A total of 233 003 patients (97 617 [41.9%] female patients; mean [SD] age, 58.0 [12.1] years) across 6565 dialysis facilities met inclusion criteria. Participants included 70 062 Black patients (30.1%), 42 820 Hispanic patients (18.4%), 105 368 White patients (45.2%), and 14 753 patients (6.3%) who identified as another race or ethnicity (eg, American Indian or Alaskan Native, Asian, Native Hawaiian or Pacific Islander, and multiracial). Overall 19 766 patients (8.5%) were wait-listed for kidney transplant within 1 year of dialysis initiation, and 48 846 patients (21.0%) had commercial insurance. Among dialysis facilities, the mean (SD) commercial payer mix was 21.3% (15.6 percentage points). eFigure 2 in Supplement 1 shows the distribution of commercial payer mix across facilities. A total of 623 facilities (9.5%) cared for no commercially insured patients, and 38 facilities (0.6%) cared for exclusively commercially insured patients. Facility commercial payer mix was categorized into quartiles: Q1, 0% to 10.5%; Q2, 10.6% to 19.2%; Q3, 19.3% to 29.6%; and Q4, 29.7% to 100%.

### Patient and Facility Characteristics by Quartile of Facility Commercial Payer Mix

Table 1 shows the patient and facility characteristics by quartile of facility commercial payer mix. Compared with Q1 dialysis facilities, Q4 facilities were more likely to be in urban areas (991 facilities [75.5%] vs 1227 facilities [90.4%]), more likely affiliated with large chain corporations (947 facilities [72.1%] vs 1053 facilities [77.5%]), had a higher DFC rating (mean [SD], 3.6 [1.1] stars vs 3.8 [1.0] stars), were more likely for-profit (1151 facilities [87.7%] vs 1240 facilities [91.3%]), and had a higher patient-to-social worker ratio (mean [SD], 57.7 [33.8] patients per social worker vs 64.6 [34.9] patients per social worker). Compared with patients in Q1 facilities, patients in Q4 facilities were less likely to be Hispanic (14 291 patients [28.9%] vs 6348 patients [11.9%]), more likely employed (3952 patients [8.0%] vs 11 210 patients [21.0%]), from counties with lower SVI (mean [SD], 0.7 [0.2] vs 0.5 [0.2]), and more likely to be receiving peritoneal dialysis (2562 patients [5.2%] vs 8569 patients [16.0%]). There were no systematic trends in comorbidity burden between groups.

**Table 1.** Facility and Patient Characteristics of the Study Cohort, by Quartile of Dialysis Facility Commercial Payer Mix

Characteristic	No. (%)					SMD, Q4 vs Q1^a^
	Total	Dialysis facility commercial payer mix quartile				
		1 (lowest)	2	3	4 (highest)	
**Dialysis facility level (2017)**						
No.	5550	1313	1437	1442	1358	NA
Size						
Large (>25 stations)	753 (13.6)	149 (11.3)	253 (17.6)	197 (13.7)	154 (11.3)	0.04
Medium (11-25 stations)	4192 (75.5)	983 (74.9)	1089 (75.8)	1122 (77.8)	998 (73.5)	
Small (≤10 stations)	605 (10.9)	181 (13.8)	95 (6.6)	123 (8.5)	206 (15.2)	
Geographical location						
Micropolitan	635 (11.4)	181 (13.8)	194 (13.5)	162 (11.2)	98 (7.2)	0.42
Rural	355 (6.4)	141 (10.7)	93 (6.5)	88 (6.1)	33 (2.4)	
Urban	4560 (82.2)	991 (75.5)	1150 (80.0)	1192(82.7)	1227 (90.4)	
Chain affiliation						
Independent	702 (12.6)	220 (16.8)	165 (11.5)	147 (10.2)	170 (12.5)	0.13
Large chain	4248 (76.5)	947 (72.1)	1130 (78.6)	1118 (77.5)	1053 (77.5)	
Small or regional chain	600 (10.8)	146 (11.1)	142 (9.9)	177 (12.3)	135 (9.9)	
Facility CMS 5-star ranking						
1	116 (2.1)	45 (3.4)	24 (1.7)	26 (1.8)	21 (1.5)	0.18
2	409 (7.4)	121 (9.2)	85 (5.9)	87 (6.0)	116 (8.5)	
3	1943 (35.0)	467 (35.6)	536 (37.3)	517 (35.9)	423 (31.1)	
4	1584 (28.5)	322 (24.5)	419 (29.2)	437 (30.3)	406 (29.9)	
5	1498 (27.0)	358 (27.3)	373 (26.0)	375 (26.0)	392 (28.9)	
For-profit	5000 (90.1)	1151 (87.7)	1305 (90.8)	1304 (90.4)	1240 (91.3)	0.12
Patient-to-nurse ratio, mean (SD)	16.3 (8.5)	15.5 (8.3)	17.6 (8.8)	16.3 (7.7)	15.7 (8.8)	0.02
Patient-to-social worker ratio, mean (SD)	64.6 (33.2)	57.7 (33.8)	68.2 (30.9)	67.5 (32.2)	64.6 (34.9)	0.20
**Patient characteristics 2013-2017**						
No.	233 003	49 474	65 834	64 208	53 487	NA
Sex						
Female	97 617 (41.9)	20 737 (41.9)	28 133 (42.7)	26 719 (41.6)	22 028 (41.2)	0.01
Male	135 386 (58.1)	28 737 (58.1)	37 701 (57.3)	37 489 (58.4)	31 459 (58.8)	
Age, mean (SD), y	58.0 (12.1)	58.0 (12.1)	58.0 (12.2)	58.0 (12.1)	58.0 (12.1)	0.003
Race and ethnicity						
Black, non-Hispanic	70 062 (30.1)	13 837 (28.0)	20 780 (31.6)	19 244 (30.0)	16 201 (30.3)	0.45
Hispanic	42 820 (18.4)	14 291 (28.9)	12 933 (19.6)	9248 (14.4)	6348 (11.9)	
Other^b^	14 753 (6.3)	3391 (6.9)	3440 (5.2)	4077 (6.3)	3845 (7.2)	
White, non-Hispanic	105 368 (45.2)	17 955 (36.3)	28 681 (43.6)	31 639 (49.3)	27 093 (50.7)	
Insurance type						
Commercial	48 846 (21.0)	2777 (5.6)	9855 (15.0)	15 525 (24.2)	20 689 (38.7)	0.91
Medicaid	68 939 (29.6)	18 980 (38.4)	21 636 (32.9)	17 653 (27.5)	10 670 (19.9)	
Medicare	81 675 (35.1)	17 926 (36.2)	24 228 (36.8)	22 761 (35.4)	16 760 (31.3)	
Other	17 462 (7.5)	5982 (12.1)	4884 (7.4)	3979 (6.2)	2617 (4.9)	
Uninsured	16 081 (6.9)	3809 (7.7)	5231 (7.9)	4290 (6.7)	2751 (5.1)	
Employment status						
Employed	31 873 (13.7)	3952 (8.0)	7070 (10.7)	9641 (15.0)	11 210 (21.0)	0.43
Not employed	83 215 (35.7)	21 687 (43.8)	24 355 (37.0)	21 563 (33.6)	15 610 (29.2)	
Retired	117 915 (50.6)	23 835 (48.2)	34 409 (52.3)	33 004 (51.4)	26 667 (49.9)	
Social Vulnerability Index, mean (SD)^c^	0.6 (0.2)	0.7 (0.2)	0.7 (0.2)	0.6 (0.2)	0.5 (0.2)	0.74
Initial modality type						
Home hemodialysis	531 (0.2)	80 (0.2)	94 (0.1)	143 (0.2)	214 (0.4)	0.36
In-center	209 810 (90.0)	46 832 (94.7)	60 892 (92.5)	57 382 (89.4)	44 704 (83.6)	
Peritoneal dialysis	22 662 (9.7)	2562 (5.2)	4848 (7.4)	6683 (10.4)	8569 (16.0)	
Primary cause of kidney failure						
Cystic kidney	4199 (1.8)	720 (1.5)	1067 (1.6)	1210 (1.9)	1202 (2.2)	0.14
Diabetes	125 423 (53.8)	27 208 (55.0)	35 708 (54.2)	34 773 (54.2)	27 734 (51.9)	
Glomerulonephritis	17 078 (7.3)	2893 (5.8)	4382 (6.7)	5100 (7.9)	4703 (8.8)	
Hypertension	65 032 (27.9)	14 479 (29.3)	18 845 (28.6)	17 014 (26.5)	14 694 (27.5)	
Other	18 931 (8.1)	3706 (7.5)	5236 (8.0)	5434 (8.5)	4555 (8.5)	
Urologic	2340 (1.0)	468 (0.9)	596 (0.9)	677 (1.1)	599 (1.1)	
Comorbidities						
Congestive heart failure	60 969 (26.2)	13 190 (26.7)	17 796 (27.0)	16 596 (25.8)	13 387 (25.0)	0.04
Coronary artery disease	27 320 (11.7)	5620 (11.4)	7781 (11.8)	7368 (11.5)	6551 (12.2)	0.03
Stroke	16 722 (7.2)	3299 (6.7)	4986 (7.6)	4702 (7.3)	3735 (7.0)	0.01
Peripheral vascular disease	21 567 (9.3)	4909 (9.9)	6301 (9.6)	5546 (8.6)	4811 (9.0)	0.03
Hypertension	206 798 (88.8)	43 604 (88.1)	58 547 (88.9)	56 970 (88.7)	47 677 (89.1)	0.03
Diabetes, insulin-dependent	106 664 (45.8)	22 474 (45.4)	30 842 (46.8)	29 478 (45.9)	23 870 (44.6)	0.02
Chronic obstructive pulmonary disease	18 100 (7.8)	3585 (7.2)	5566 (8.5)	5189 (8.1)	3760 (7.0)	0.01
Tobacco	16 021 (6.9)	3067 (6.2)	4875 (7.4)	4666 (7.3)	3413 (6.4)	0.01
Nonambulatory	5077 (2.2)	1191 (2.4)	1589 (2.4)	1311 (2.0)	986 (1.8)	0.04

Abbreviations: NA, not applicable; SMD, standardized mean difference.

^a^ Measured as the magnitude of the difference of a given variable between the lowest and highest quartile of facility commercial payer mix; values of 0.2 to 0.5 are generally considered small to modest, and values greater than 0.8 are considered large.

^b^ Other refers to American Indian or Alaskan Native, Asian, Native Hawaiian or Pacific Islander, and multiracial.

^c^ Social Vulnerability Index ranges from 0 (least vulnerable) to 1 (most vulnerable).

### Association of Individual Patients’ Commercial Insurance Status With Wait-Listing for Kidney Transplant

The unadjusted incidence of wait-listing was 2.7-fold higher in patients with commercial vs noncommercial insurance (18.6%; 95% CI, 18.1%-18.8%; vs 6.8%; 95% CI, 6.7%-6.9%). After adjustment for patient and facility characteristics (but not facility-level commercial payer mix), individual patients with commercial insurance had 86% higher hazards of wait-listing compared with publicly insured patients (hazard ratio [HR], 1.86; 95% CI, 1.80-1.93; *P* < .001).

### Association of Facility Commercial Payer Mix With Wait-Listing for Kidney Transplant

**Figure 1.**
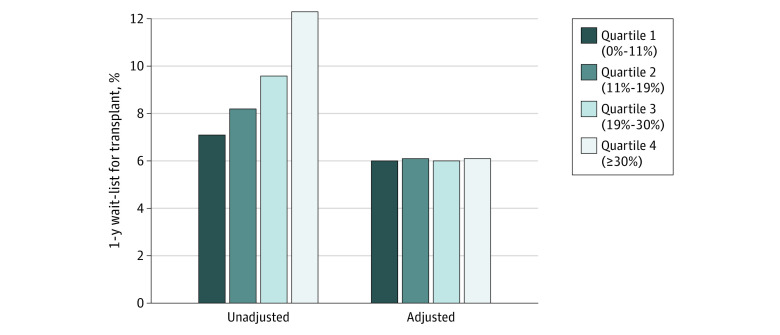
One-Year Incidence of Kidney Transplant Wait-Listing, by Quartile of Dialysis Facility Commercial Payer Mix The adjusted numbers are from the fully adjusted Cox model censoring for death within the first year, including covariates for patient (demographics, insurance status, employment, neighborhood Social Vulnerability Index, dialysis modality, cause of kidney failure, medical comorbidities, year of dialysis initiation) and facility (location, size, for-profit status, large chain affiliation) characteristics.

Table 2 shows the association of facility-level commercial payer mix with 1-year wait-listing for transplant using Cox regression models. Full models are presented in eTables 1 through 3 in Supplement 1. Although there was an initial association of payer mix with outcome in the unadjusted model and in an adjusted model excluding individual patients’ insurance status, once patient-level insurance status was accounted for, there was no significant independent association of facility payer mix with wait-listing (Q4 vs Q1: HR, 1.02; 95% CI, 0.95-1.09; *P* = .60). Figure 1 shows unadjusted and fully adjusted incidence of wait-listing by quartile of facility commercial payer mix. Adjusted incidence of wait-listing was similar across quartiles.

**Table 2.** Association of Dialysis Facility Commercial Payer Mix With Wait-Listing for Kidney Transplantation Within 1 Year of Dialysis Initiation

Commercial payer mix, quartile	Factors included in covariate adjustment					
	Unadjusted		Patient and facility factors, excluding individual patient insurance^a^		Patient and facility factors, including individual patient insurance^a^	
	HR (95% CI)	*P* value	HR (95% CI)	*P* value	HR (95% CI)	*P* value
1 (Lowest)	1 [Reference]	NA	1 [Reference]	-	1 [Reference]	NA
2	1.17 (1.09-1.26)	<.001	1.10 (1.04-1.18)	.003	1.02 (0.96-1.09)	.51
3	1.38 (1.29-1.48)	<.001	1.15 (1.08-1.23)	<.001	1.01 (0.94-1.08)	.82
4 (Highest)	1.79 (1.67-1.91)	<.001	1.25 (1.17-1.34)	<.001	1.02 (0.95-1.09)	.60

Abbreviations: HR, hazard ratio; NA, not applicable.

^a^ Patient factors included demographics, race and ethnicity, employment status, neighborhood-level Social Vulnerability Index, dialysis modality type, cause of kidney failure, medical comorbidities, and year of dialysis initiation. Facility factors included size, location, corporate chain affiliation, and for-profit status. Full model results are presented in eTable 1 and eTable 2 in Supplement 1.

### Association of Facility Commercial Payer Mix With Wait-Listing for Kidney Transplant, by Individual Insurance Status

**Figure 2.**
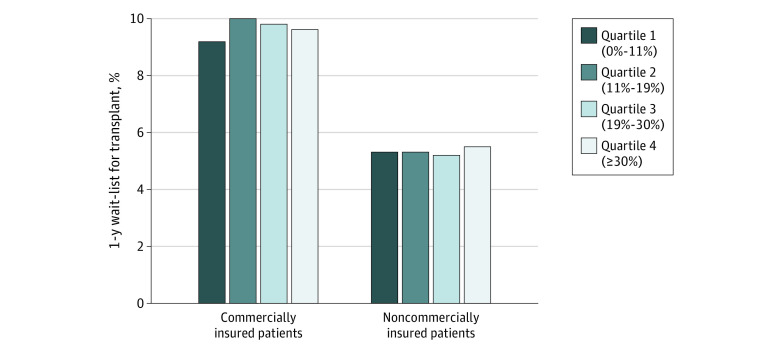
One-Year Incidence of Kidney Transplant Wait-Listing, by Quartile of Dialysis Facility Commercial Payer Mix and Individual Insurance Type The adjusted numbers are from the fully adjusted Cox model censoring for death within the first year and using an interaction term for payer mix by individual insurance status and including covariates for patient (demographics, insurance status, employment, neighborhood Social Vulnerability Index, dialysis modality, cause of kidney failure, medical comorbidities, year of dialysis initiation) and facility (location, size, for-profit status, large chain affiliation) characteristics. Of 233 003 patients in the full cohort, 48 846 (21%) had commercial insurance and 184 157 (79%) had noncommercial insurance.

Next, we assessed the association of facility payer mix with outcome among patients with commercial vs noncommercial insurance using an interaction term of payer mix quartile by insurance status. There was no independent association of facility payer mix with outcome in either subset of commercially vs noncommercially insured patients (Table 3). As shown in Figure 2, adjusted incidence of wait-listing was overall higher for commercially insured patients than noncommercially insured, but within these groups, the incidence of wait-listing was similar across payer mix quartiles.

**Table 3.** Association of Dialysis Facility Commercial Payer Mix With Wait-Listing for Kidney Transplantation Within 1 Year of Dialysis Initiation, by Patient Insurance Status

Commercial payer mix, quartile	Association of facility commercial payer mix^a^			
	Commercially insured patients only (n = 48 846)		Noncommercially insured patients only (n = 184 157)	
	HR (95% CI)	*P* value	HR (95% CI)	*P* value
1 (Lowest)	1 [Reference]	NA	1 [Reference]	-
2	1.08 (0.96-1.22)	.18	1.01 (0.94-1.08)	.87
3	1.06 (0.95-1.19)	.31	0.99 (0.92-1.06)	.78
4 (Highest)	1.04 (0.93-1.17)	.47	1.04 (0.96-1.12)	.35

Abbreviations: HR, hazard ratio; NA, not applicable.

^a^ Covariates used for adjustment included patient factors (demographics, race and ethnicity, employment status, neighborhood-level Social Vulnerability Index, dialysis modality type, cause of kidney failure, medical comorbidities, and year of dialysis initiation) and facility factors (size, location, corporate chain affiliation, and for-profit status). Full model results are presented in eTable 3 in Supplement 1.

### Sensitivity Analyses

We repeated the analyses (using fully adjusted models) in a few subgroups. Among 158 937 patients aged 65 years or younger, commercial payer mix was not associated with wait-listing (Q4 vs Q1: HR, 1.01; 95% CI, 0.95-1.09; *P* = .70; Q3 vs Q1: HR = 1.01; 95% CI, 0.95-1.08; *P* = .72; Q2 vs Q1: HR, 1.04; 95% CI, 0.97-1.11; *P* = .28). Among 141 689 patients aged 65 years or younger without contraindications to transplant, payer mix was not associated with wait-listing (Q4 vs Q1: HR, 1.02; 95% CI, 0.95-1.10; *P* = .59; Q3 vs Q1: HR, 1.02; 95% CI, 0.95-1.09; *P* = .64; Q2 vs Q1: HR = 1.03; 95% CI, 0.96-1.10; *P* = .43). Finally, the results were unchanged (null association for payer mix) among subsets of nonprofit, independent, and small or regional chain facilities.

## Discussion

In this cohort study using a national sample of patients with incident ESKD receiving chronic dialysis, higher dialysis facility commercial payer mix was not independently associated with higher rates of wait-listing for transplant. At the patient level, incidence of wait-listing was higher for commercially insured patients compared with publicly insured patients. Although higher payer mix facilities had higher unadjusted incidence of wait-listing, attributable to higher proportions of commercially insured patients, when adjusting for individual insurance, there was no significant independent association of payer mix with access to transplant. There remained no significant association of payer mix with wait-listing within groups of commercially vs noncommercially insured patients (ie, no effect modification). These findings suggest that access to kidney transplant may be influenced by a beneficial association of commercial insurance for individual patients or to unobserved differences in the commercially insured patient population rather than facility-specific differences in performance by high commercial payer mix dialysis facilities.

These findings are timely, given recent controversial practices by dialysis corporations in allegedly steering patients toward commercial insurance. With a higher dialysis reimbursement rate by commercial insurers relative to Medicare or Medicaid,^4^ for-profit dialysis corporations have been suspected of steering Medicare- or Medicaid-eligible patients toward commercial insurance in the individual marketplace, even subsidizing patients’ premiums, thus generating higher revenue in return.^5,16^ This practice poses potential risks to patients, including disruption in coverage, higher out-of-pocket costs for nondialysis health care services, and potentially hindering the transplant evaluation process.^5^ Additionally, the practice of subsidizing premiums risks harming patients in the posttransplant setting when these subsidies are likely to disappear.^5^ Although we did not study this practice of switching from public to commercial insurance, our findings suggest commercial insurance may be directly beneficial to patients and improve their access to transplantation, at least in this cohort of patients with commercial insurance at dialysis initiation. These findings warrant further study to identify and optimize socioeconomic drivers of access to care for patients with ESKD, either through adequate insurance coverage or other social determinants of health.

There are several potential explanations for the association of commercial insurance with improved access to transplant. Commercial insurance itself may improve access to transplant for patients. Prior studies have shown a link between commercial insurance and increased kidney transplant evaluation and wait-listing.^8,10^ This is attributed to easier ability to complete the required steps in the transplant evaluation process^9,17,18^ but may also reflect socioeconomic differences in commercially insured populations. Dialysis facilities in neighborhoods with higher socioeconomic status have higher referral and transplantation rates,^2,19^ and patients from more socially vulnerable communities are less likely to receive a living donor kidney transplant.^20^ We adjusted for race and ethnicity, employment status, and county-level SVI in this analysis; nevertheless, there may be socioeconomic differences that are unaccounted for in our study. Furthermore, although we did not find payer mix to have an independent association with transplant wait-listing at the facility level, we did find that for-profit facilities had lower incidences of wait-listing, consistent with prior studies.^8^ suggesting that facility characteristics related to organizational structure or financial operations may still have a role in influencing transplant access, even independent of an individual’s insurance status.

This study has implications for health policy. Dialysis facilities with high commercial payer mix generate more revenue than facilities with predominantly publicly insured patients and can likely afford additional staffing to facilitate transplant education and referrals. Yet, we did not find improved facility wait-listing rates independent of the association of an individual’s insurance. Thus, the additional revenue generated by facilities with high commercial payer mix did not translate with improved access to transplant for the patients in those facilities. However, direct financial incentives tied to transplant-related metrics may provide more of an impetus for dialysis facilities to invest in improving transplant access, and this is the intent of recently implemented CMS pay-for-performance initiatives in ESKD, such as the ESRD Treatment Choices model^3^ and the ESRD Quality Incentive Program.^21^ The ESRD Treatment Choices model was implemented in January 2021 and incentivizes participating dialysis facilities to increase transplantation by adjusting reimbursement according to a facility’s wait-listing and living donor transplant rates. As of 2022, the ESRD Quality Incentive Program is now using percentage of prevalent patients wait-listed as a performance metric. The effects of these programs on transplant access needs monitoring.

Insurance coverage and dialysis reimbursement will remain a salient issue with the recent expansion of MA eligibility. Patients with ESKD previously had limited ability to enroll in MA, but as of January 2021, all patients with ESKD are eligible to enroll in MA. These plans are managed by commercial insurers but can have more restricted health care networks, which may pose barriers to transplant evaluation. By December 2021, the proportion of patients with ESKD enrolled in MA increased 50%,^22^ and further increases are expected^23^; the resulting impact on access to transplantation must be monitored.

### Limitations

This study has some limitations. This is an observational study and therefore reports associations but cannot prove causality. Patient insurance is reported on CMS Form 2728 by patient report or by the clinician and is thus subject to misclassification, yet this form remains the most commonly used for ascertaining such demographic information at dialysis initiation.^12^ We adjusted for many patient and facility characteristics in our models, but there are likely still unmeasured confounders, particularly socioeconomic, that could explain our findings. Additionally, dialysis facilities cannot directly add patients to transplant wait-lists, but rather, are tasked with referring patients to transplant centers. Referral data are not available in the USRDS. Nevertheless, 1-year incidence of wait-listing is a useful proxy for early referral and a metric of access to transplantation.^13^ Although this study focuses on dialysis facilities, the transplant centers play an undeniable role in ensuring access, and the impact of insurance and payer mix among transplant centers warrants exploration in future work. Lastly, our analysis is limited to chronic dialysis and does not capture patients with chronic kidney disease who may benefit from preemptive kidney transplantation.

## Conclusions

In this cohort study among patients newly initiated to receive chronic dialysis, commercially insured patients were more likely to be wait-listed for transplant within 1 year of starting dialysis, but receiving care in a facility with higher commercial payer mix was not independently associated with incidence of kidney transplant wait-listing. Thus, the association of commercial insurance with access to kidney transplant was primarily at the individual patient level, whether due to the insurance coverage itself or socioeconomic differences of the commercially insured ESKD population, whereas the commercial payer mix of a dialysis facility was not associated with a patient’s chance of being added to a waiting list. The landscape of insurance coverage and reimbursement for dialysis continues to evolve and has implications for patients’ ability to access kidney transplantation. Policies affecting dialysis reimbursement should continue to be monitored for their downstream impact on patient access to kidney transplantation.

## Notes

Supplement 1. **eFigure 1.** Inclusion and Exclusion Criteria for the Study Cohort **eFigure 2. **Distribution of Commercial Payer Mix Across 6565 US Dialysis Facilities **eTable 1.** Results of Multivariable Cox Regression Model of Wait-listing for Kidney Transplantation Within 1 Year of Dialysis Initiation Without Inclusion of Patient-Level Insurance **eTable 2.** Results of Fully Adjusted Multivariable Cox Regression Model of Wait-listing for Kidney Transplantation Within 1 Year of Dialysis Initiation With Inclusion of Patient-Level Insurance **eTable 3.** Results of Multivariable Cox Regression Model of Wait-listing for Kidney Transplantation Within 1 Year of Dialysis Initiation With Interaction Term for Payer Mix by Individual Patient Insurance

Supplement 2.
Data Sharing Statement
