# Supplementary material for: The Association of Dialysis Facility Payer Mix With Access to Kidney Transplantation
Source: JAMA Netw Open. 2023 Jul 11;6(7):e2322803. doi: 10.1001/jamanetworkopen.2023.22803 (PMC10336615; doi:10.1001/jamanetworkopen.2023.22803)
Supplement: Supplement 1. — eFigure 1. Inclusion and Exclusion Criteria for the Study Cohort eFigure 2. Distribution of Commercial Payer Mix Across 6565 US Dialysis Facilities eTable 1. Results of Multivariable Cox Regression Model of Wait-listing for Kidney Transplantation Within 1 Year of Dialysis Initiation Without Inclusion of Patient-Level Insurance eTable 2. Results of Fully Adjusted Multivariable Cox Regression Model of Wait-listing for Kidney Transplantation Within 1 Year of Dialysis Initiation With Inclusion of Patient-Level Insurance eTable 3. Results of Multivariable Cox Regression Model of Wait-listing for Kidney Transplantation Within 1 Year of Dialysis Initiation With Interaction Term for Payer Mix by Individual Patient Insurance [file jamanetwopen-e2322803-s001.pdf]

Supplemental Online Content

Cron DC, Tsai TC, Patzer RE, Husain SA, Xiang L, Adler JT. The association of dialysis facility payer mix with access to kidney transplantation. *JAMA Netw Open*. 2023;6(7):e2322803. doi:10.1001/jamanetworkopen.2023.22803

eFigure 1. Inclusion and Exclusion Criteria for the Study Cohort

eFigure 2. Distribution of Commercial Payer Mix Across 6565 US Dialysis Facilities

eTable 1. Results of Multivariable Cox Regression Model of Wait-listing for Kidney Transplantation Within 1 Year of Dialysis Initiation Without Inclusion of Patient-Level Insurance

eTable 2. Results of Fully Adjusted Multivariable Cox Regression Model of Wait-listing for Kidney Transplantation Within 1 Year of Dialysis Initiation With Inclusion of Patient-Level Insurance

eTable 3. Results of Multivariable Cox Regression Model of Wait-listing for Kidney Transplantation Within 1 Year of Dialysis Initiation With Interaction Term for Payer Mix by Individual Patient Insurance

This supplemental material has been provided by the authors to give readers additional information about their work.

© 2023 Cron DC et al. *JAMA Network Open.*

eFigure 1. Inclusion and exclusion criteria for the study cohort. Pre-emptively waitlisted patients were added to the waitlist prior to starting dialysis and thus were excluded.

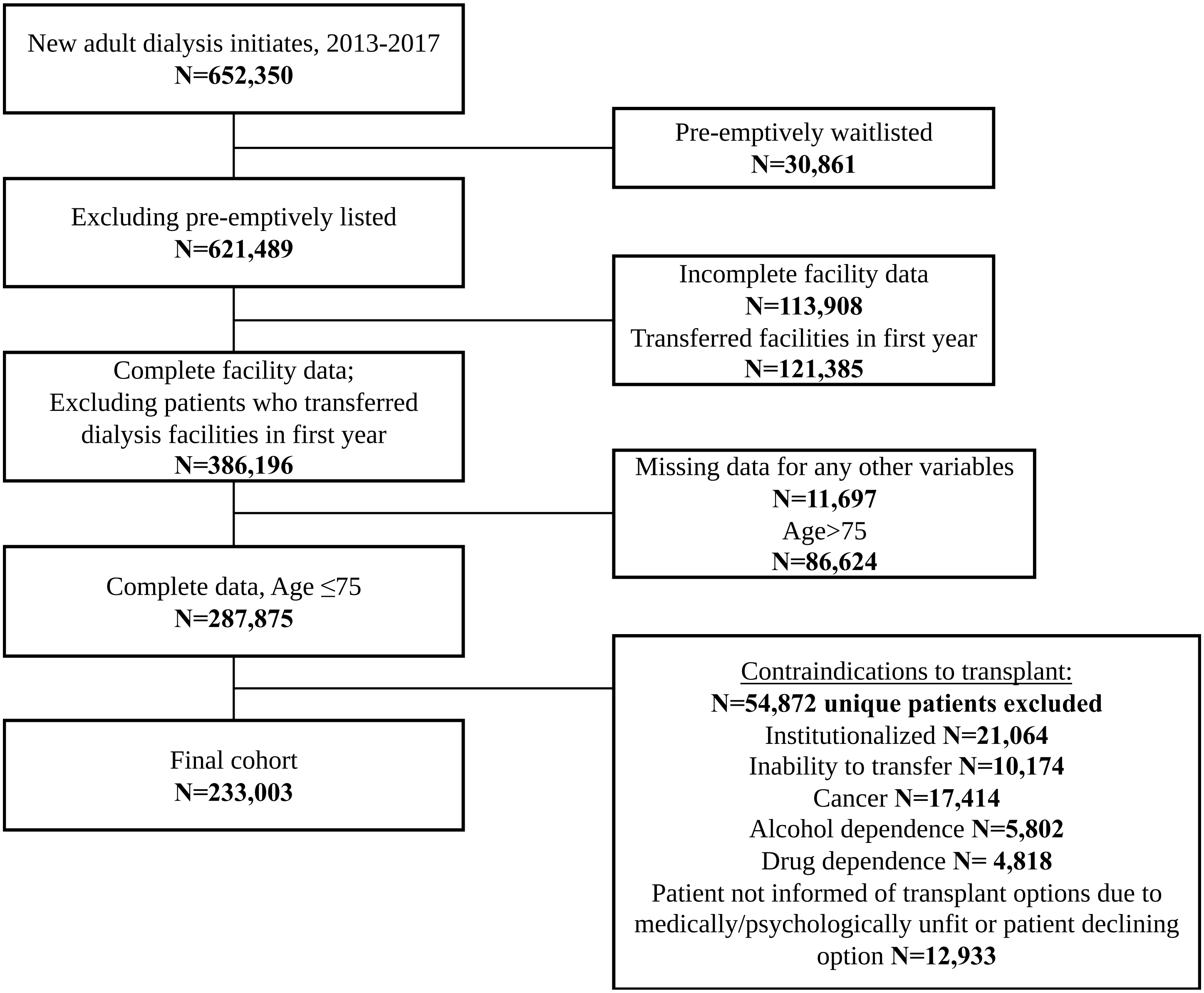

© 2023 Cron DC et al. *JAMA Network Open.*

eFigure 2. Distribution of commercial payer mix across 6,565 U.S. dialysis facilities. Mean commercial payer mix was 21.2%, and 623 facilities (9.5%) cared for zero commercially insured patients, while 38 facilities (0.6%) cared for exclusively commercially insured patients.

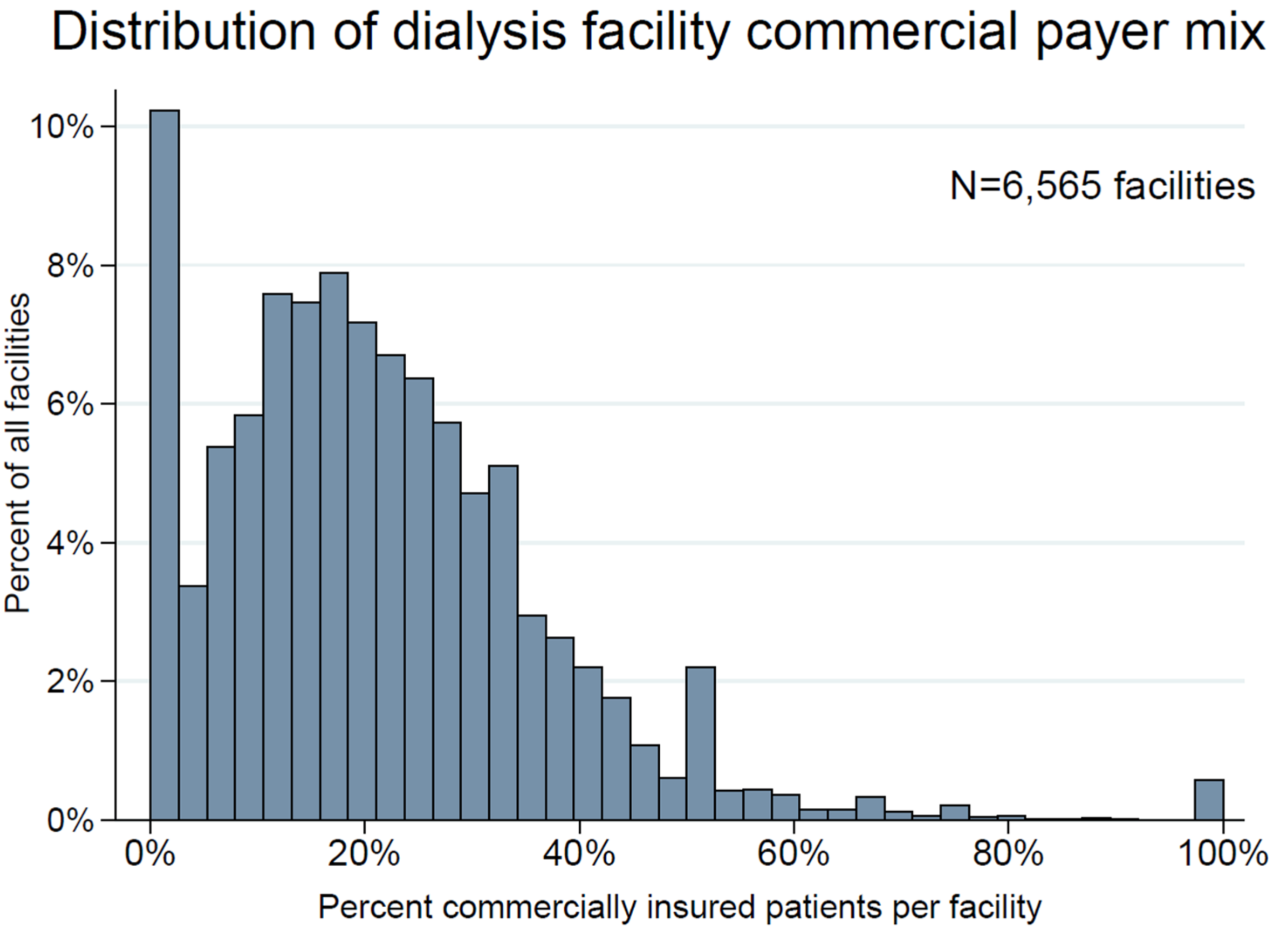

© 2023 Cron DC et al. *JAMA Network Open.*

eTable 1. Results of multivariable Cox regression model of waitlisting for kidney transplantation within 1 year of dialysis initiation (*without* inclusion of patient-level insurance).

Variables	*HR*	*95% C.I.*	*P-value*
**Commercial payer mix variable** (quartiles)			
Q1 – lowest (Ref.)	1	-	-
Q2	1.10	(1.04 - 1.18)	0.003
Q3	1.15	(1.08 - 1.23)	<0.001
Q4 – highest	1.25	(1.17 - 1.34)	<0.001
**Facility characteristics**			
Facility size			
Small (≤10 stations; Ref.)	1		
Medium (11-25 stations)	1.04	(0.96 - 1.13)	0.33
Large (>25 stations)	0.94	(0.86 - 1.03)	0.20
Geographical location			
Rural (Ref.)	1		
Micropolitan	1.10	(0.97 - 1.25)	0.12
Urban	1.26	(1.13 - 1.41)	<0.001
Dialysis chain affiliation			
Independent (Ref.)	1		
Large chain	1.00	(0.94 - 1.07)	0.96
Small or regional chain	0.95	(0.87 - 1.04)	0.28
For-profit facility	0.81	(0.75 - 0.88)	<0.001
**Patient characteristics**			
Sex			
Female (Ref.)	1		
Male	1.30	(1.26 - 1.33)	<0.001
Age (per year)	0.97	(0.97 - 0.97)	<0.001
Race/ethnicity			
White non-Hispanic (Ref.)	1		
Black non-Hispanic	0.84	(0.81 - 0.87)	<0.001
Hispanic	1.07	(1.02 - 1.12)	0.004
Other	1.14	(1.06 - 1.23)	0.001
Employment status			
Not employed (Ref.)	1		
Employed	1.89	(1.82 - 1.97)	<0.001
Retired	1.00	(0.96 - 1.04)	0.93
Social Vulnerability Index (SVI - per 0.1 increase)	0.98	(0.97 - 0.99)	<0.001
Initial modality type			
In-center (Ref.)	1		
Home hemodialysis	1.47	(1.15 - 1.89)	0.002
Peritoneal dialysis	2.04	(1.96 - 2.14)	<0.001
Primary cause of renal failure			
Diabetes (Ref.)	1		
Cystic kidney	1.78	(1.65 - 1.93)	<0.001
Glomerulonephritis	1.47	(1.40 - 1.54)	<0.001
Hypertension	1.00	(0.96 - 1.04)	0.88
Other	0.95	(0.90 - 1.02)	0.14
Urologic	0.86	(0.75 - 0.99)	0.04

© 2023 Cron DC et al. *JAMA Network Open.*

Comorbidities			
Congestive heart failure	0.60	(0.57 - 0.63)	<0.001
Coronary artery disease	0.89	(0.83 - 0.94)	<0.001
Stroke	0.70	(0.65 - 0.76)	<0.001
Peripheral vascular disease	0.69	(0.64 - 0.75)	<0.001
Hypertension	1.15	(1.10 - 1.21)	<0.001
Diabetes - insulin-dependent	0.91	(0.88 - 0.95)	<0.001
Chronic obstructive pulmonary disease	0.47	(0.42 - 0.52)	<0.001
Non-ambulatory	0.27	(0.21 - 0.35)	<0.001
Tobacco	0.45	(0.42 - 0.49)	<0.001
Year of dialysis initiation			
2013 (Ref.)	1		
2014	0.86	(0.83 - 0.90)	<0.001
2015	0.76	(0.72 - 0.79)	<0.001
2016	0.71	(0.68 - 0.75)	<0.001
2017	0.63	(0.60 - 0.66)	<0.001

Commercial payer mix is primary exposure variable. HR=hazard ratio. C.I.=confidence interval. Social vulnerability index is a continuous variable ranging from 0 (least vulnerable) to 1 (most vulnerable). ESRD network region was also included in the model for adjustment but not shown here for brevity.

© 2023 Cron DC et al. *JAMA Network Open.*

eTable 2. Results of <u>fully adjusted</u> multivariable Cox regression model of waitlisting for kidney transplantation within 1 year of dialysis initiation (*with* inclusion of patient-level insurance).

Variables	*HR*	*95% C.I.*	*P-value*
**<u>Commercial payer mix variable</u>** (quartiles)			
Q1 – lowest (Ref.)	1		
Q2	1.02	(0.96 - 1.09)	0.51
Q3	1.01	(0.94 - 1.08)	0.82
Q4 – highest	1.02	(0.95 - 1.09)	0.60
**<u>Facility characteristics</u>**			
Facility size			
Small (≤10 stations; Ref.)	1		
Medium (11-25 stations)	1.03	(0.95 - 1.12)	0.44
Large (>25 stations)	0.94	(0.86 - 1.03)	0.21
Geographical location			
Rural (Ref.)	1		
Micropolitan	1.12	(0.99 - 1.27)	0.08
Urban	1.27	(1.14 - 1.42)	<0.001
Dialysis chain affiliation			
Independent (Ref.)	1		
Large chain	0.99	(0.93 - 1.06)	0.84
Small or regional chain	0.94	(0.86 - 1.03)	0.21
For-profit facility	0.81	(0.75 - 0.88)	<0.001
**<u>Patient characteristics</u>**			
Sex			
Female (Ref.)	1		
Male	1.28	(1.24 - 1.32)	<0.001
Age (per year)	0.97	(0.97 - 0.97)	<0.001
Race/ethnicity			
White non-Hispanic (Ref.)	1		
Black non-Hispanic	0.85	(0.82 - 0.89)	<0.001
Hispanic	1.12	(1.07 - 1.17)	<0.001
Other	1.17	(1.09 - 1.27)	<0.001
Insurance status			
Non-commercial (Ref.)	1		
Commercial	1.86	(1.79 - 1.92)	<0.001
Employment status			
Not employed (Ref.)	1		
Employed	1.46	(1.40 - 1.52)	<0.001
Retired	1.05	(1.00 - 1.09)	0.03
Social Vulnerability Index (SVI - per 0.1 increase)	0.98	(0.97 - 0.99)	<0.001
Initial modality type			
In-center (Ref.)	1		
Home hemodialysis	1.38	(1.08 - 1.77)	0.01
Peritoneal dialysis	1.98	(1.90 - 2.08)	<0.001
Primary cause of renal failure			
Diabetes (Ref.)	1		
Cystic kidney	1.76	(1.62 - 1.90)	<0.001
Glomerulonephritis	1.44	(1.37 - 1.51)	<0.001

© 2023 Cron DC et al. *JAMA Network Open.*

Hypertension	1.02	(0.98 - 1.06)	0.40
Other	0.95	(0.89 - 1.01)	0.12
Urologic	0.88	(0.76 – 1.02)	0.09
Comorbidities			
Congestive heart failure	0.61	(0.58 - 0.64)	<0.001
Coronary artery disease	0.89	(0.83 - 0.94)	<0.001
Stroke	0.71	(0.66 - 0.77)	<0.001
Peripheral vascular disease	0.70	(0.65 - 0.76)	<0.001
Hypertension	1.15	(1.09 - 1.20)	<0.001
Diabetes - insulin-dependent	0.91	(0.87 - 0.94)	<0.001
Chronic obstructive pulmonary disease	0.48	(0.43 - 0.53)	<0.001
Non-ambulatory	0.28	(0.22 - 0.36)	<0.001
Tobacco	0.47	(0.43 - 0.51)	<0.001
Year of dialysis initiation			
2013 (Ref.)	1		
2014	0.87	(0.83 - 0.90)	<0.001
2015	0.76	(0.72 - 0.79)	<0.001
2016	0.72	(0.69 - 0.75)	<0.001
2017	0.64	(0.61 - 0.67)	<0.001

Commercial payer mix is primary exposure variable. HR=hazard ratio. C.I.=confidence interval. Social vulnerability index is a continuous variable ranging from 0 (least vulnerable) to 1 (most vulnerable). ESRD network region was also included in the model for adjustment but not shown here for brevity.

© 2023 Cron DC et al. *JAMA Network Open.*

eTable 3. Results of multivariable Cox regression model of waitlisting for kidney transplantation within 1 year of dialysis initiation (with interaction term for payer mix by individual patient insurance)

Variables	*HR*	*95% C.I.*	*P-value*
**Commercial payer mix variable** (quartiles)			
Commercially insured patients			
Q1 – lowest (Ref.)	1		
Q2	1.08	(0.96 - 1.22)	0.18
Q3	1.06	(0.95 - 1.19)	0.31
Q4 – highest	1.04	(0.93 - 1.17)	0.47
Non-commercially insured patients			
Q1 – lowest (Ref.)	1		
Q2	1.01	(0.94 - 1.08)	0.87
Q3	0.99	(0.92 - 1.06)	0.78
Q4 – highest	1.04	(0.96 - 1.12)	0.35
**Facility characteristics**			
Facility size			
Small (≤10 stations; Ref.)	1		
Medium (11-25 stations)	1.03	(0.95 - 1.12)	0.45
Large (>25 stations)	0.94	(0.86 - 1.03)	0.20
Geographical location			
Rural (Ref.)	1		
Micropolitan	1.12	(0.99 - 1.27)	0.08
Urban	1.27	(1.14 - 1.42)	<0.001
Dialysis chain affiliation			
Independent (Ref.)	1		
Large chain	0.99	(0.93 - 1.06)	0.85
Small or regional chain	0.95	(0.87 - 1.03)	0.21
For-profit facility	0.81	(0.75 - 0.88)	<0.001
**Patient characteristics**			
Sex			
Female (Ref.)	1		
Male	1.28	(1.24 - 1.32)	<0.001
Age (per year)	0.97	(0.97 - 0.97)	<0.001
Race/ethnicity			
White non-Hispanic (Ref.)	1		
Black non-Hispanic	0.86	(0.82 - 0.89)	<0.001
Hispanic	1.12	(1.07 - 1.17)	<0.001
Other	1.18	(1.09 - 1.27)	<0.001
Insurance status			
Non-commercial (Ref.)	1		
Commercial	1.78	(1.59 - 1.99)	<0.001
Employment status			
Not employed (Ref.)	1		
Employed	1.46	(1.40 - 1.52)	<0.001
Retired	1.05	(1.00 - 1.09)	0.03
Social Vulnerability Index (SVI - per 0.1 increase)	0.98	(0.97 - 0.99)	<0.001
Initial modality type			
In-center (Ref.)	1		

© 2023 Cron DC et al. *JAMA Network Open.*

Home hemodialysis	1.38	(1.08 - 1.77)	0.009
Peritoneal dialysis	1.99	(1.90 - 2.08)	<0.001
Primary cause of renal failure			
Diabetes (Ref.)	1		
Cystic kidney	1.76	(1.63 - 1.90)	<0.001
Glomerulonephritis	1.44	(1.37 - 1.51)	<0.001
Hypertension	1.02	(0.98 - 1.06)	0.40
Other	0.95	(0.89 - 1.01)	0.12
Urologic	0.88	(0.76 – 1.02)	0.09
Comorbidities			
Congestive heart failure	0.61	(0.58 - 0.64)	<0.001
Coronary artery disease	0.89	(0.83 - 0.94)	<0.001
Stroke	0.71	(0.66 - 0.77)	<0.001
Peripheral vascular disease	0.70	(0.65 - 0.76)	<0.001
Hypertension	1.15	(1.09 - 1.20)	<0.001
Diabetes - insulin-dependent	0.91	(0.87 - 0.94)	<0.001
Chronic obstructive pulmonary disease	0.48	(0.43 - 0.53)	<0.001
Non-ambulatory	0.28	(0.22 - 0.36)	<0.001
Tobacco	0.47	(0.43 - 0.51)	<0.001
Year of dialysis initiation			
2013 (Ref.)	1		
2014	0.87	(0.83 - 0.90)	<0.001
2015	0.76	(0.72 - 0.79)	<0.001
2016	0.72	(0.69 - 0.75)	<0.001
2017	0.64	(0.61 - 0.67)	<0.001

Commercial payer mix is primary exposure variable. This model included an interaction term of facility payer mix by patient insurance status, and this was used to calculate the association of payer mix within commercially vs. non-commercially insured patients. HR=hazard ratio. C.I.=confidence interval. Social vulnerability index is a continuous variable ranging from 0 (least vulnerable) to 1 (most vulnerable). ESRD network region was also included in the model for adjustment but not shown here for brevity.

© 2023 Cron DC et al. *JAMA Network Open.*
