# Supplementary material for: The Association of Dialysis Facility Payer Mix With Access to Kidney Transplantation
Source: JAMA Netw Open. 2023 Jul 11;6(7):e2322803. doi: 10.1001/jamanetworkopen.2023.22803 (PMC10336615; doi:10.1001/jamanetworkopen.2023.22803)
Supplement: Supplement 2. — Data Sharing Statement [file jamanetwopen-e2322803-s002.pdf]

Data Sharing Statement

Cron. The Association of Dialysis Facility Payer Mix with Access to Kidney Transplantation. *JAMA Netw Open*. Published July 11, 2023. doi:10.1001/jamanetworkopen.2023.22803

Data
Data available: No

Additional Information
Explanation for why data not available: The dataset is publicly available; cohort code can be provided.
